# Supplementary material for: DMRforPairs: identifying Differentially Methylated Regions between unique samples using array based methylation profiles
Source: BMC Bioinformatics. 2014 May 15;15:141. doi: 10.1186/1471-2105-15-141 (PMC4046028; doi:10.1186/1471-2105-15-141)
Supplement: Additional file 2 — DMRforPairs output for the comparison of A431-MCF7 and NA17018-NA17105. Please start from the HTML files in each folder. Available via the BMC Bioinformatics website. [file 1471-2105-15-141-S2.zip › 1394847754114233_MOESM2_ESM/A431_MCF7/figures/1288.pdf]

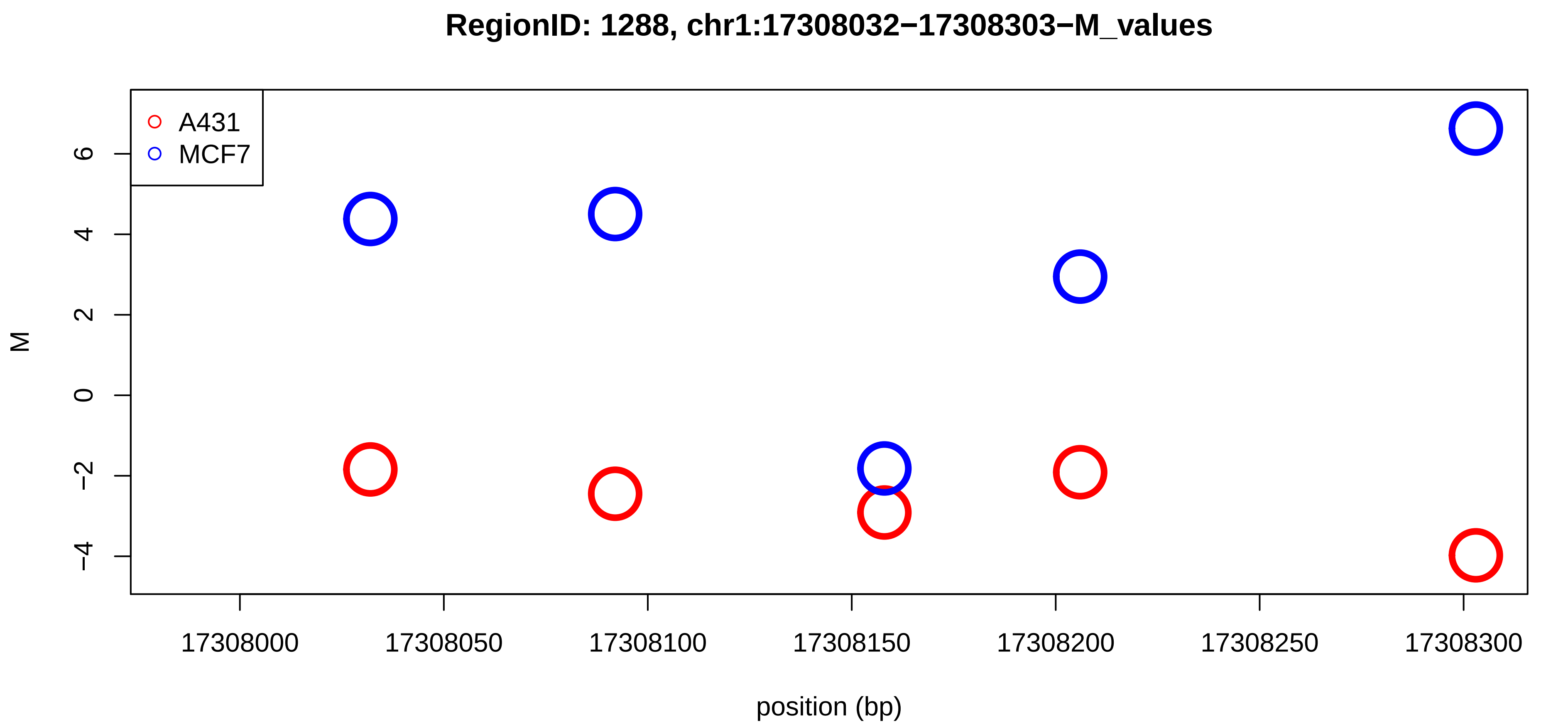

RegionID: 1288, chr1:17308032−17308303−M_values
A431
MCF7
M
6
4
2
0
−2
−4
17308000
17308050
17308100
17308150
17308200
17308250
17308300
position (bp)

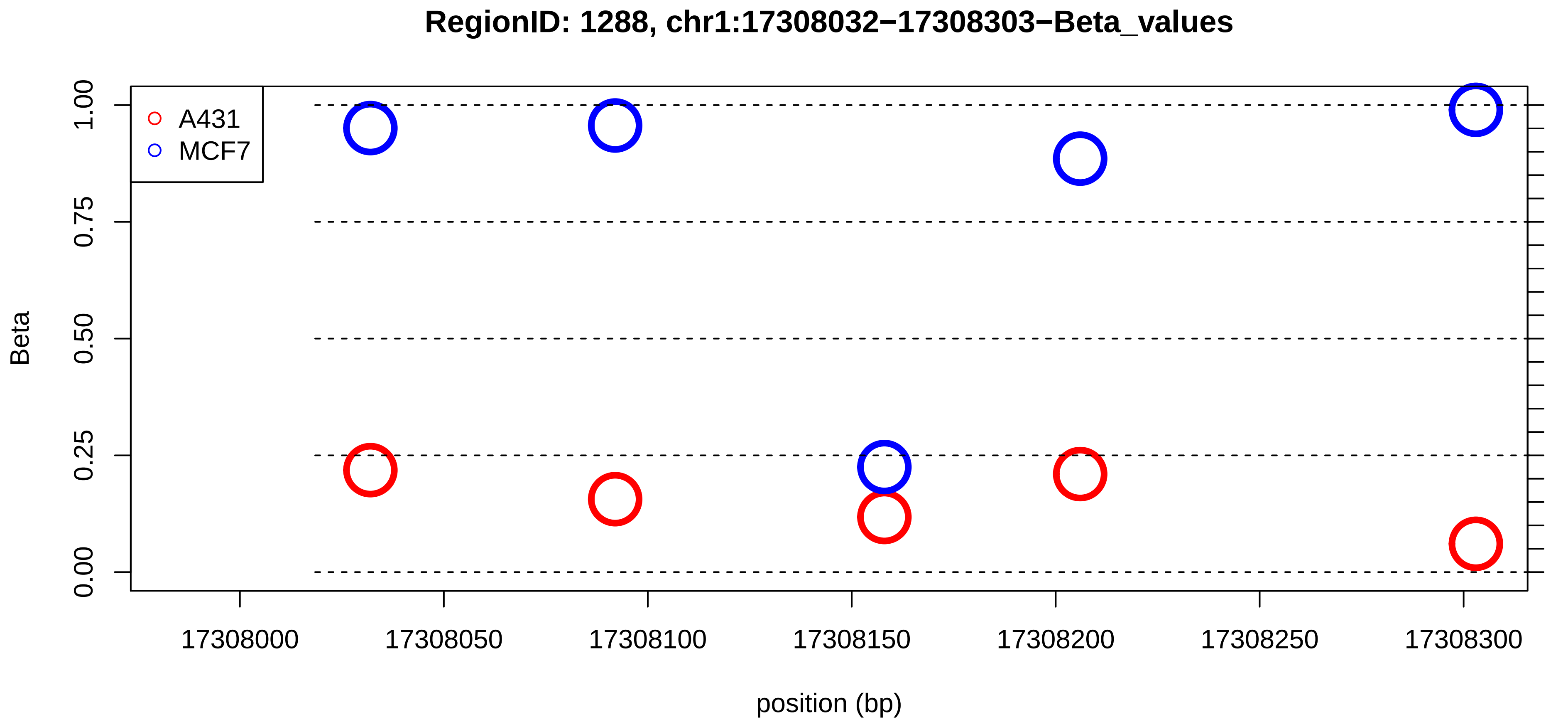

RegionID: 1288, chr1:17308032−17308303−Beta_values
A431
MCF7
Beta
1.00
0.75
0.50
0.25
0.00
17308000
17308050
17308100
17308150
17308200
17308250
17308300
position (bp)

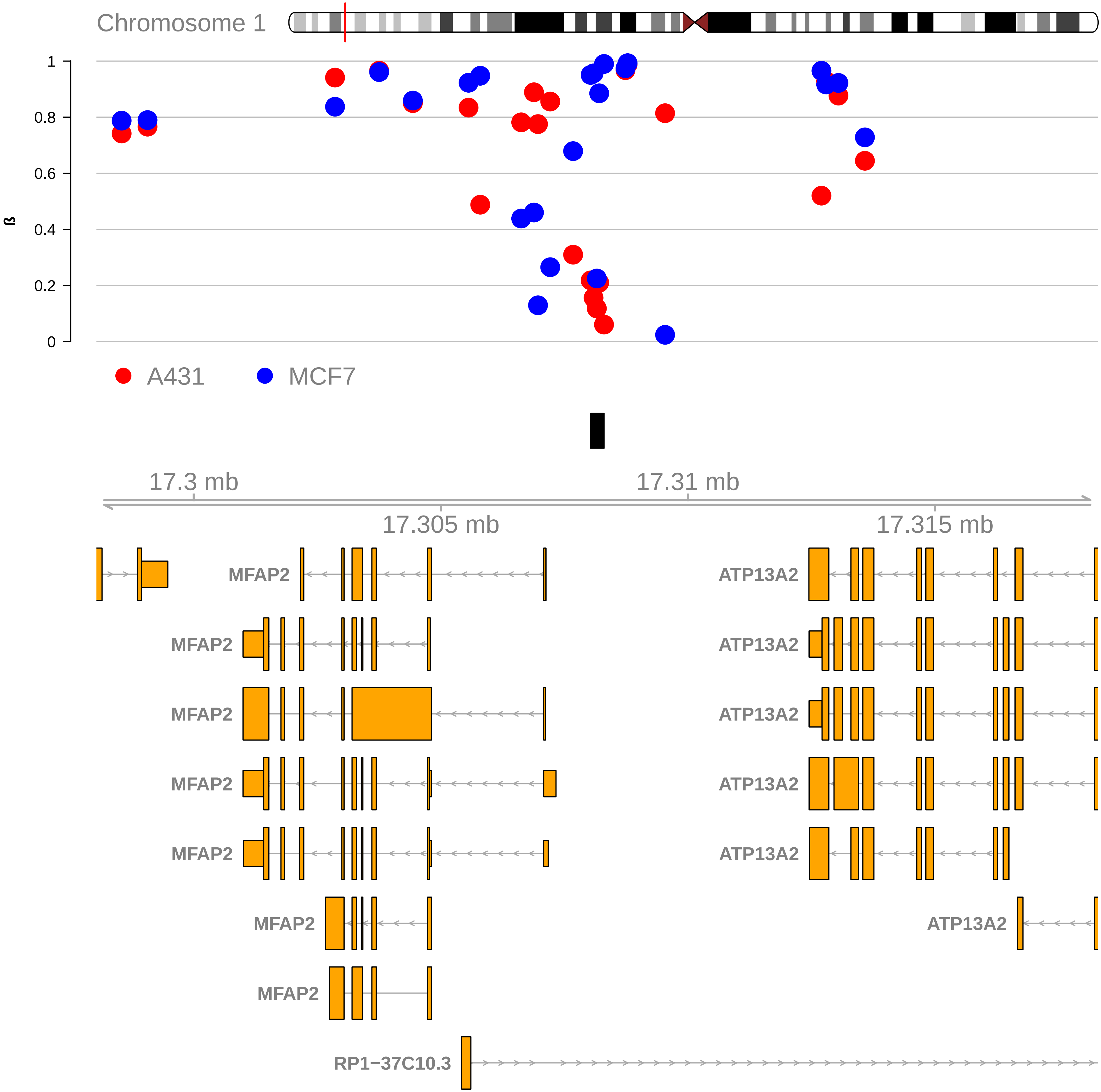

Chromosome 1
1
0.8
0.6
0.4
0.2
0
β
A431
MCF7
17.3 mb
17.31 mb
17.305 mb
17.315 mb
MFAP2
MFAP2
MFAP2
MFAP2
MFAP2
MFAP2
MFAP2
ATP13A2
ATP13A2
ATP13A2
ATP13A2
ATP13A2
ATP13A2
RP1−37C10.3
